# Ultrafast and highly sensitive infrared photodetectors based on two-dimensional oxyselenide crystals

**DOI:** 10.1038/s41467-018-05874-2

**Published:** 2018-08-17

**Authors:** Jianbo Yin, Zhenjun Tan, Hao Hong, Jinxiong Wu, Hongtao Yuan, Yujing Liu, Cheng Chen, Congwei Tan, Fengrui Yao, Tianran Li, Yulin Chen, Zhongfan Liu, Kaihui Liu, Hailin Peng

**Affiliations:** 1 Center for Nanochemistry, Beijing Science and Engineering Centre for Nanocarbons, Beijing National Laboratory for Molecular Sciences, College of Chemistry and Molecular Engineering, Peking University, Beijing, 100871 China; 2 Academy for Advanced Interdisciplinary Studies, Peking University, Beijing, 100871 China; 3 State Key Laboratory for Mesoscopic Physics, School of Physics, Peking University, Beijing, 100871 China; 4 National Laboratory of Solid-State Microstructures, College of Engineering and Applied Sciences, and Collaborative Innovation Center of Advanced Microstructures, Nanjing University, Nanjing, 210093 China; 5 Clarendon Laboratory, Department of Physics, University of Oxford, Parks Road, Oxford, OX1 3PU UK

**Keywords:** Two-dimensional materials, Two-dimensional materials

## Abstract

Infrared light detection and sensing is deeply embedded in modern technology and human society and its development has always been benefitting from the discovery of various photoelectric materials. The rise of two-dimensional materials, thanks to their distinct electronic structures, extreme dimensional confinement and strong light–matter interactions, provides a material platform for next-generation infrared photodetection. Ideal infrared detectors should have fast respond, high sensitivity and air-stability, which are rare to meet at the same time in one two-dimensional material. Herein we demonstrate an infrared photodetector based on two-dimensional Bi_2_O_2_Se crystal, whose main characteristics are outstanding in the whole two-dimensional family: high sensitivity of 65 AW^−1^ at 1200 nm and ultrafast photoresponse of ~1 ps at room temperature, implying an intrinsic material-limited bandwidth up to 500 GHz. Such great performance is attributed to the suitable electronic bandgap and high carrier mobility of two-dimensional oxyselenide.

## Abstract (web summary)

Two-dimensional (2D) bismuth oxyselenide crystals with suitable electronic band-gap and ultrahigh carrier mobility enable near-infrared photodetection. Here, the authors report an infrared photodetector based on 2D-bismuth oxyselenide with high responsivity, ultrafast photoresponse of ~ 1 ps at room temperature and a detectable frequency limit of up to 500 GHz.

## Introduction

The discovery of various types of materials is the main driving force for the development of infrared (IR) photodetection beyond silicon’s detection wavelength limit (around 1100 nm). After century’s efforts, different bulk materials with high quantum efficiency and tunable band gaps in the IR region, such as III–V and II–VI compounds of HgCdTe, InGaAs, InSb, GaAs/AlGaAs quantum wells, and InAs/GaSb super-lattices, have been discovered^1^. Currently, an important trend in IR detection is the combination of IR sensing materials with silicon readout circuit, enabling larger number of pixels, higher frame rates and more complicated on-chip signal-processing functions. To this end, great efforts have been devoted to finding various IR-sensitive materials with compatibility to silicon readout circuits, such as platinum silicide (PtSi)^2^, black silicon^3^, and quantum dots^4,5^. However, the sensitivity and intrinsic response speed of these materials still have lots of room for the improvement.

The recent rise of two-dimensional (2D) layered materials has opened up more possibilities for high-performing photodetection, thanks to their extreme dimensional confinement in the thickness and strong light–matter interactions in 2D plane^6–11^. In addition, 2D layered materials have excellent mechanical properties and dangling-bond-free interlayers, which allow for an easy processing of atomically thin layers into focal plan arrays (FPA) and compatibility with readout circuits^12,13^. However, 2D layered materials has not yet shown simultaneous high sensitivity and fast photoelectric response in detecting IR light. For example, graphene shows high-speed photoresponse^14^ but very low intrinsic sensitivity less than tens of mAW^−1^ ^9^. Transition metal dichalcogenides (TMDs) usually have too large band gaps to detect IR light. Few-layer black phosphorus films exhibit promising infrared photodetection due to the fast carrier dynamics arising from its substantial mobility and moderate bandgap^15–17^. However, its environmental instability and incompatibility with large-scale fabrication processes have hindered its potential applications^18^. Therefore, identifying air-stable 2D layered materials for highly sensitive and high-speed IR detection is highly motivated.

Herein we report high-performing IR photodetectors based on an air-stable 2D oxyselenide crystals at room temperature. The prototype devices of 2D Bi_2_O_2_Se demonstrate a very high sensitivity of 65 AW^−1^ at 1200 nm and an ultrafast photoresponse ~1 ps, implying an ultrahigh material-limited photodetection bandwidth up to 500 GHz^8,14,19–21^. Such an outstanding performance should originate from Bi_2_O_2_Se crystal’s appropriate band gap and high carrier mobility. In combination with excellent flexibility (strain up to 1%), high stability (at least months in air) and the capability of mass production, 2D Bi_2_O_2_Se detectors hold promise in low-cost infrared imaging, high-speed photodetection and flexible biosensor operating at room temperature.

## Results

### Band structure and photodetector of 2D Bi_2_O_2_Se

**Fig. 1:**
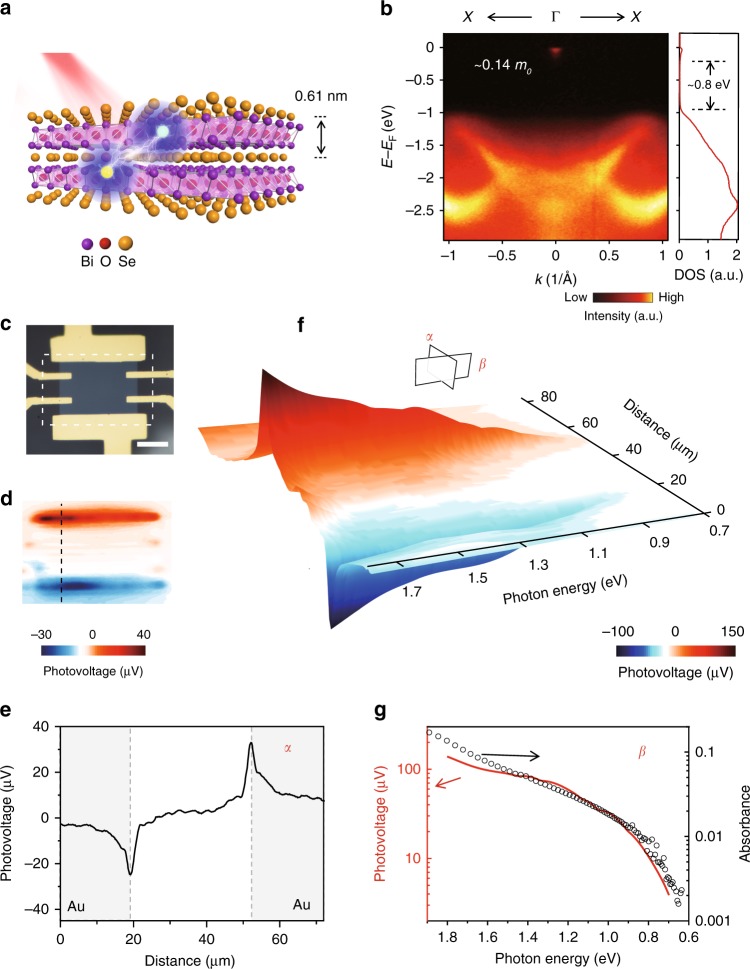
Photodetectors based on 2D Bi_2_O_2_Se. **a** Illustration of photodetector based on 2D Bi_2_O_2_Se crystal, with schematic crystal structure of alternatively stacked (Bi_2_O_2_)_n_ and Se_n_ layers. The layer thickness is 0.61 nm. **b** Electronic structure of Bi_2_O_2_Se observed by ARPES, which shows an indirect band gap of ~0.8 eV and small effective mass of ~0.14 *m*_0_. The spectra under *E*–*E*_F_ = −1 eV and around *E*–*E*_F_ = 0eV correspond to valence and conduction band, respectively. *E*_F_ refers to Fermi level, so only tiny conduction band structure under Femi level is observed here. The directions of ARPES mapping are along X–Γ–X directions. The right panel is the corresponding density of states (DOS), which is calculated by integrating signals of the left panel along momentum space. **c** Optical image of Bi_2_O_2_Se device with thickness of ~10 nm and ~16 layers, scale bar 20 μm. **d** Scanning photovoltage image of the dotted rectangle area of device in **c**. The 1200 nm laser with power of 150 μW is focused and scanned on the device, while the net photovoltages are recorded as function of laser positions. The photovoltages are measured without external bias. **e** Line-scanning of photovoltage along dotted line in **d**. The curve corresponds to the α-plane cut of **f**. The two peaks correspond with Bi_2_O_2_Se-metal junctions. **f** Spectrogram composed of photovoltage line scans at different incident photon energies (wavelength of incident light). The incident photon numbers are kept still during the measurement, and the incident power is 100 μW at 1200 nm. **g** Photovoltage and absorbance of Bi_2_O_2_Se as function of incident photon energy. Red solid line shows photovoltage trace, which corresponds to β-plane cut of the spectrogram. Black circles show the absorbance of Bi_2_O_2_Se film with ~10 nm in thickness

Very recently, Bi_2_O_2_Se emerged as a promising 2D layered material with excellent air stability and high-mobility semiconducting behavior^22^. As shown in Fig. 1a, Bi_2_O_2_Se is a layered oxyselenide that consists of alternatively stacked Bi_2_O_2_ and Se layers with a layer thickness of 0.61 nm. The unique lattice of Bi_2_O_2_Se gives rise to an appropriate band gap of ~0.8 eV and relatively small electron effective mass of ~0.14 *m*_0_, which are revealed by both theoretical calculations^22^ and angle-resolved photoemission spectroscopy (ARPES, see Materials and methods), as shown in Fig. 1b. The layered nature also makes it ideal for fabricating electronic devices down to few atomic layers, which has shown high Hall mobility (29,000 cm^2^ V^−1^ s^−1^ at 1.9 K and 450 cm^2^ V^−1^ s^−1^ at room temperature^22^, also shown in Supplementary Fig. 1) and high current on/off ratio of >10^6^ with almost ideal subthreshold swing of 65 mV dec^−1^ at room temperature^22^. Such high mobility facilitates the photocarrier extraction process, which, in combination with the appropriate band gap, underlies high sensitivity and fast response as introduced below, making 2D Bi_2_O_2_Se a promising material for IR photodetection.

To understand the optoelectronic properties of 2D Bi_2_O_2_Se crystals, we firstly synthesized individual Bi_2_O_2_Se nanosheets with a domain size up to tens of μm and a thickness down to few layers on a mica substrate by chemical vapor deposition (CVD, see Methods)^22,23^, and then fabricated a photodetection device on it (Fig. 1c). We measured the photoresponse of 2D Bi_2_O_2_Se crystal without applying external bias to guarantee the intrinsic photovoltaic response, and found a broadband response with the spectrum from visible to 1700 nm. In detail, we revealed photovoltage distribution by scanning a laser beam (150 μW, 1200 nm, and about 1.5 μm in spot size) over the device (Fig. 1c), and recording the photovoltages accordingly. As shown in Fig. 1d, e, prominent photovoltage signals were generated at two Bi_2_O_2_Se-metal junctions with opposite polarities, which result from photocarriers separation process—electron–hole pairs are separated to opposite directions by the symmetric build-in electrical fields at the two junctions. This phenomenon implies that the photocurrent generation is highly dependent on the photocarrier separation process, although other processes also play important roles, such as photocarrier excitation.

To study the photocarrier excitation process of Bi_2_O_2_Se, we recorded photocurrents while changing the wavelengths of incident light and showed photovoltage spectrum in Fig. 1f, g. The spectrum extends to ~1700 nm (0.73 eV), which agrees in a reasonable accuracy with the band gap value (~0.8 eV) observed by ARPES and optical absorption in Fig. 1g (right *y* axis). Such agreement confirms that the photocarrier excitation originates from interband transition. In addition, as the photon energy increases, the photocurrent increases in the same trend with the optical absorption, as shown by the black circle in Fig. 1g, implying that photocurrent generation is also subject to the photocarrier generation process. This increasing trend is in agreement with the change of density of states (DOS) as shown in Fig. 1b—away from band gap, both conduction and valence bands have increasing DOS, favoring the interband transition.

### Photoresponsivity under photoconductivity regime

**Fig. 2:**
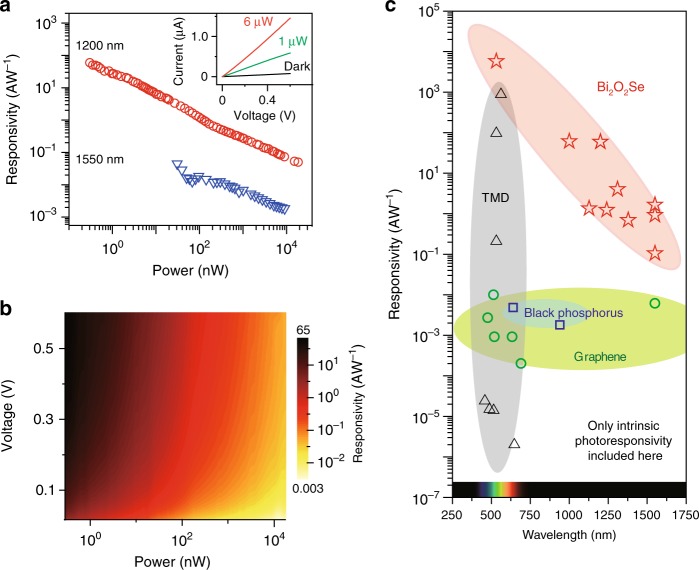
High photoresponsivity of 2D Bi_2_O_2_Se photodetector. **a** Photoresponsivity under 0.6 V bias at wavelengths of 1200 nm and 1500 nm. **b** Dependence of photoresponsivity with incident power and voltage bias at 1200 nm wavelength. **c** Comparison of photodetectors based on Bi_2_O_2_Se, graphene, black phosphorus, and transition metal dichalcogenides (TMDs)^8,9,21,24–29,36^. Note that the data only includes the photocurrent generation through excitation of interband transitions without further treatment such as adding waveguide structure or plasmonic structure

To evaluate the photosensitivity and detectivity of Bi_2_O_2_Se detector, we applied source–drain bias on the device to create a photoconductivity regime. In this regime, photocurrent *I*_ph_ is defined as difference of drain currents between dark and illuminated states (inset of Fig. 2a), and responsivity (*R*) is calculated by equation:

1 $$R = I_{{\mathrm{ph}}}/P$$

where *P* denotes incident power. To quantify the photosensitivity of 2D Bi_2_O_2_Se devices, we summarized the responsivities at 1200 nm as scatter plot in Fig. 2a, and color chart in Fig. 2b with different biases. Both figures show a responsivity of 65 AW^−1^ at incident power of 100 pW scale, implying the capability of Bi_2_O_2_Se in detecting weak infrared signals, which is very important for practical applications. This excellent performance can be further evidenced by high sensitivities along the whole spectrum from visible to IR regime, such as 5800 AW^−1^ at 532 nm, 4 AW^−1^ at 1310 nm (Supplementary Fig. 2) and 0.1 AW^−1^ responsivity at 1550 nm (Fig. 2a, c). In comparison with other 2D materials, which show low intrinsic sensitivities in infrared spectral range (not including further treatment for enhancing photocurrent, such as utilizing detect adding waveguide structure or plasmonic structure), 2D Bi_2_O_2_Se exhibits high sensitivity in an extremely broad spectral region of 300–1700 nm (Fig. 2c), much better than other 2D materials such as graphene and TMD^8,9,21,24–29^. The high sensitivities here are attributed to the existence of photoconductive gain, which is usually introduced by localized states (trap states) either inside 2D materials or at material-substrate interface. Under illumination, these localized states could trap one type of photocarriers (electrons or holes), leaving the other type of photocarriers free and circulating with prolonged life time (*τ*_trap_), as shown in Supplementary Fig. 3. Under external source–drain voltage, these free photocarriers circulate across the channel with time duration of *τ*_transit_. When *τ*_transit_ is smaller than *τ*_trap_, one free photocarrier from one photoexcitation event contributes more than one transit event in photocurrent generation, which introduces photoconductive gain with value of *τ*_trap_/*τ*_transit_^30^. This gain mechanism only prevails with the presence of external bias, as it needs external electrical field for driving one photocarrier to contribute multiple times in photocurrent measurement. The photoconductive gain is a typical feature in photodetectors of low-dimensional materials^31^ and is further explained in Supplementary note 1 and Supplementary Fig. 3, 4.

To compare 2D Bi_2_O_2_Se with commercial detection materials, we calibrate the detectivity (D^*^) of the 2D Bi_2_O_2_Se detectors by using equation:

2 $$D^ \ast = R \times \sqrt A /S_n$$

where *R* is the responsivity, *A* is the device area of ~50 × 30 µm^2^, and *S*_*n*_ is the noise spectral density at specific frequency (8.5 × 10^−11^ A Hz^−1/2^ at 1 Hz, See Supplementary Fig. 5). The detectivity of 2D Bi_2_O_2_Se detector has maximum of 3.0 × 10^9^ Jones at 1 Hz and 1200 nm wavelength at room temperature. Such detectivity is promising as a prototype device based on CVD-grown samples, given that the commercial infrared detectors usually range from 10^9^ to 10^12^ Jones at room temperature.

### Photoresponse time measured by time-resolved photocurrent spectroscopy

**Fig. 3:**
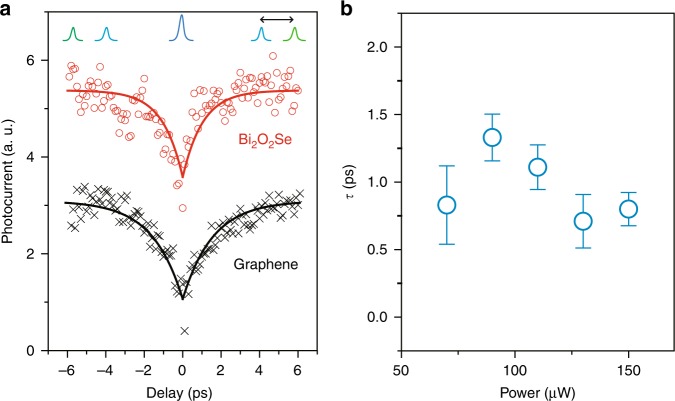
Time-resolved photocurrent spectroscopy of 2D Bi_2_O_2_Se photodetector. **a** Photocurrent as a function of delay time between two ultrafast pulses (~100 fs) at zero bias voltage. The red circles and black crosses are data of Bi_2_O_2_Se and graphene photodetector, while solid lines are exponential fitting. The blue and green curves in the top of the figure represent pump and probe laser pulses, while the black arrow shows the delay time between these two pulses. When delay time is too short, the excitation of the first pulse does not have enough time to turn into current before it meets the excitation of the second pulse, which induces sublinearity in photocurrent and reaches a dip at zero delay. When delay time is long enough, excitations from the two pulses perform independently and result in largest voltage, which is used for normalization value in this chart. **b** Response time (*τ*) of Bi_2_O_2_Se photodetector measured at different incident pump power. The response time is defined as time constant *τ* in exponential fitting of the photocurrent–delay curve by equation 3. Error bars here describe standard errors of the fitting parameter

In addition to the sensitivity, the photoresponse time is another important criterion that determines the material-limited intrinsic bandwidth of a photodetector^8,14,19–21,32^ (Note that the bandwidth of a photodetector can be limited by many other factors, such as parasitic capacitance, device geometry). To probe that, we performed time-resolved photocurrent spectroscopy^14,19,20,33,34^ at zero source–drain bias (see Methods), in which a pump and a probe laser beam (820 nm, pulse width 100 fs) were focused on the same spot at metal–Bi_2_O_2_Se junction. As shown in a typical measurement in Fig. 3a, when pump and probe pulses overlap with zero time delay the photocurrent reaches minimum due to the sublinear power-dependent photocurrent. With time-delay increasing from zero, the photocurrent increases from minimum correspondingly. Thus, the dip in photocurrent curve directly reflects the detector’s ability to distinguish two temporally nearby pulse excitation. It also represents how fast the photocarriers from the first pulse’s excitation can be extracted before the second pulse’s arrival. With exponential fitting of the rising curve, we found the response time (*τ*) is ~1 ps, as shown in Fig. 3b, translating into material-limited bandwidth of *f* = 0.55/*τ*~500 GHz^14,19,20,33,34^. Such response time is comparable to the reference graphene sample of ~1.3 ps, as shown by black curve in Fig. 3a, and significantly shorter than other 2D material as summarized in Supplementary Fig. 6. We believe that the ultrafast response of Bi_2_O_2_Se originates from the high electron mobility of Bi_2_O_2_Se, enabling the fast photocarrier drift out of the metal–Bi_2_O_2_Se junction. This fast drift allows a fast photocarrier extraction into photocurrent with ultrafast response, and is independent on the pump power, which agrees well with data in Fig. 3b (see more discussion in Supplementary note 2, Supplementary Fig. 7 and 8). Such similar response speeds can also be found in 2D Dirac materials of graphene and 3D Dirac materials of Cd_3_As_2_^14,19,35^. However, in contrast to these Dirac materials with zero band gap, layered Bi_2_O_2_Se is a 2D semiconductor with appreciable band gap and therefore demonstrates a high free carrier generation efficiency with low energy loss. In such sense, 2D Bi_2_O_2_Se should be a very promising material in high-speed infrared photodetection.

### 2D flexible Bi_2_O_2_Se photodetector arrays

**Fig. 4:**
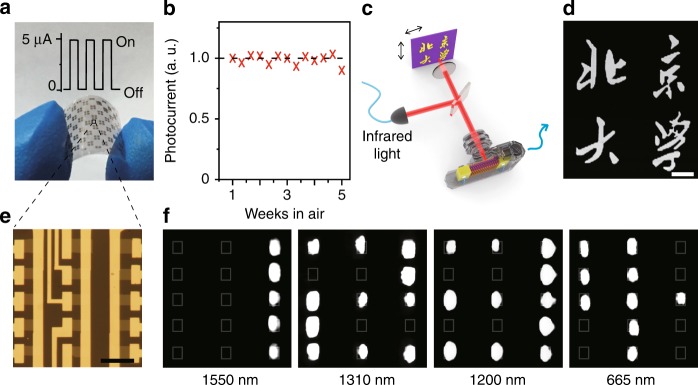
2D flexible Bi_2_O_2_Se photodetector arrays. **a** Photograph of 2D Bi_2_O_2_Se photodetectors and arrays on mica. Inset shows photoresponse of one typical photodetector when bending the substrate with strain up to 1%. The current show robust increase when illumination of 1200 nm with power of about 100 μW is on. **b** 2D Bi_2_O_2_Se photodetector maintains stable photoresponse in air for at least 5 weeks. **c** Schematic of the single-pixel imaging process. The 2D Bi_2_O_2_Se photodetector quantify the infrared reflection of the sample structure by measuring corresponding photocurrent. When the structure is scanned, the detector records the magnitudes of reflection signals and process them into image. **d** Infrared images taken by single-pixel photodetector of 2D Bi_2_O_2_Se under excitation at λ = 1150 nm. Scale bar, 100 μm. **e** Optical image of 3 × 5 multi-pixel array of 2D Bi_2_O_2_Se photodetectors. Scale bar, 10 μm. **f** The array’s photocurrent mappings are achieved by recording the photocurrents while scanning the array over a pre-focused laser beam with spot size of 1 to 2 μm. By deliberately choosing several channels in a parallel regime, photocurrents from the chosen pixels are recorded and shown in the photocurrent mapping. Here, the resulting photocurrent images with shape of 1, 2, 3, and 4 are taken under illumination of 1550 nm, 1310 nm, 1200 nm, and 665 nm light, respectively

To better understand the potential of 2D Bi_2_O_2_Se in IR sensing applications, we designed and fabricated flexible 2D Bi_2_O_2_Se photodetectors and their arrays on mica through a facile process (Fig. 4a, e and Supplementary note 3) and tested them in ambient air. The Bi_2_O_2_Se photodetector arrays show consistent photoresponse when bending the substrate with strain of up to 1% (inset of Fig. 4a), confirming that 2D Bi_2_O_2_Se photodetector can work on flexible substrates. More importantly, they are quite robust when exposed in ambient air, manifesting very stable photoresponse within at least 5 weeks (Fig. 4b), which is critical for practical applications.

Furthermore, we demonstrate the imaging capability of Bi_2_O_2_Se photodetectors by two approaches. First, single-pixel photodetector is tested to image a specific structure by scanning infrared reflection from the sample, as shown in Fig. 4c. A clear infrared image of the sample structure under 1150 nm illumination is shown in Fig. 4d, which confirms that a single photodetector of 2D Bi_2_O_2_Se can function well in a near-infrared imaging system (see Supplementary Fig. 9 and Supplementary Movie 1 for more details). Second, a 3 × 5 multi-pixel array, as shown in Fig. 4e is examined by scanning the array over a steady laser beam. As several channels are pre-picked up in a parallel regime, the photocurrents from these pixels are read out and show corresponding shapes of 1, 2, 3, and 4 under different illuminations, with wavelengths of 1550 nm, 1310 nm, 1200 nm, and 665 nm, respectively (Fig. 4f). These images from both single- and multi-pixels imply that Bi_2_O_2_Se is capable to process into FPA for multi-spectrum imaging (from visible to near infrared).

## Discussion

The imaging capability, in combination with high responsivity, ultrafast photoresponse and chemical stability, makes 2D Bi_2_O_2_Se a promising candidate for realizing ultrafast and sensitive infrared photodetectors operating at room temperature. We also note that further pathway approaching better performance is by improving crystal growth, detector geometry, and fabrication process. In this manner, concentration of localized states should be reduced and then the detector’s noise level can be lowered down. Although less localized states can also decrease photoconductive gain, the total detectivity should benefit more from low noise level. Moreover, from the view of practical application, the detector with less localized states should also show higher dynamic range which means a constant detectivity for wide incident power range. On the other hand, the carrier mobility should also increase due to less localized states. Then photocarrier collecting time in a device should decrease, which will enhance the photoresponse speed.

## Methods

### Growth and characterization of Bi_2_O_2_Se

The 2D materials of Bi_2_O_2_Se were synthesized via a previously reported chemical vapor deposition (CVD) method. The as-synthesized samples were characterized by Olympus BX51 microscope. The ARPES measurements of Bi_2_O_2_Se were carried out at beamline I05 of the Diamond Light 48 Source (DLS), with energy resolution of 20 meV and angle resolution of 0.2°.

### Device fabrication and measurement

Bi_2_O_2_Se devices are made on mica after growth and on Si/SiO_2_ (300 nm) after transfer with help of poly(methyl methacrylate). The Ti/Au (5/35 nm) electrodes were fabricated by electron-beam lithography and the following electron-beam evaporation. The photovoltage measurements were performed by a scanning photocurrent microscopy. In the set-up, Supercontinuum Laser Sources (NKT Photonic) were used as laser sources. The chopper-modulated (about 500 Hz) laser beams with spot size of 1–2 μm were focused on the device by using a 50 objective. The short-circuit photocurrents were then measured by pre-amplifier and lock-in amplifier, while the photovoltages were directly measured by lock-in amplifier. When scanning the laser spot over the device, the induced photocurrents (photovoltage) and beam positions were recorded and displayed simultaneously with the assistance of a computer, which communicated with lock-in amplifier and motorized stage (with device on it). In the photoconductivity measurement, Keithley 2400 was used to supply the source–drain bias and at the same time measure the current.

Photoresponsivity measurement is conducted under photoconductivity regime. A source–drain bias was applied to the device while the laser beam was parked still at the device. By switching on/off the laser, the drain currents at on/off states were collected. The difference of drain currents between illuminated (on) and dark (off) states is defined as photocurrent *I*_ph_. The responsivity (*R*) is calculated by equation 1, where *P* denotes incident power collected by the device.

For time-resolved measurement, two cross-polarized (minimize interference near zero delay) pulsed beams (both with pulse width of ~100 fs and wavelength of 820 nm from a Ti: sapphire 80 MHz oscillator) were focused on Bi_2_O_2_Se–metal or graphene–metal junctions. The devices are without external bias to ensure intrinsic response. One pulsed beam was chopped at frequency of 1500 Hz to generate modulated photovoltage in the device circuit. This photovoltage was sensitive to the presence of the second beam pulses, which was temporally delayed by Δ*t* through a motorized delay line. Thus, the photovoltage could be researched as function of the delay time. The sublinear photovoltage-power measurement was conducted without the presence of the second beam. The response time is defined as time constant *τ* in exponential fitting by

3 $$\frac{{I_{{\mathrm{ph}}}\left( {\Delta t} \right)}}{{I_{{\mathrm{ph}}}\left( \infty \right)}} = 1 - {\mathrm{C}} \times {\mathrm{exp}}\left( { - \Delta t/\tau } \right)$$

where Δ*t* is the absolute delay time between two pulses. *I*_ph_(Δ*t*) is the photocurrent which changes with Δ*t*, *I*_ph_(∞) is the photocurrent when delay time is infinite. C is the fitting parameter.

### Data availability

The data that support the findings of this study are available from the corresponding authors on reasonable request.

## Electronic supplementary material

Supplementary Information

Description of Additional Supplementary Files

Supplementary Movie 1
