# Author Correction: Ultrafast and highly sensitive infrared photodetectors based on two-dimensional oxyselenide crystals

**DOI:** 10.1038/s41467-019-11422-3

**Published:** 2019-07-29

**Authors:** Jianbo Yin, Zhenjun Tan, Hao Hong, Jinxiong Wu, Hongtao Yuan, Yujing Liu, Cheng Chen, Congwei Tan, Fengrui Yao, Tianran Li, Yulin Chen, Zhongfan Liu, Kaihui Liu, Hailin Peng

**Affiliations:** 1 Center for Nanochemistry, Beijing Science and Engineering Centre for Nanocarbons, Beijing National Laboratory for Molecular Sciences, College of Chemistry and Molecular Engineering, Peking University, Beijing, 100871 China; 2 Academy for Advanced Interdisciplinary Studies, Peking University, Beijing, 100871 China; 3 State Key Laboratory for Mesoscopic Physics, School of Physics, Peking University, Beijing, 100871 China; 4 National Laboratory of Solid-State Microstructures, College of Engineering and Applied Sciences, and Collaborative Innovation Center of Advanced Microstructures, Nanjing University, Nanjing, 210093 China; 5 Clarendon Laboratory, Department of Physics, University of Oxford, Parks Road, Oxford, OX1 3PU UK

**Keywords:** Two-dimensional materials, Two-dimensional materials

Correction to: *Nature Communications* 10.1038/s41467-018-05874-2, published online 17 August 2018.

The original version of this Article contained an error in Fig. 1g in which a top x-axis was shown with incorrect labels for the wavelength. The correct version of Fig. 1g, which removes this labelling, is:

which replaces the previous incorrect version:

This has been corrected in both the PDF and HTML versions of the Article.

