# Supplementary material for: Ultrafast and highly sensitive infrared photodetectors based on two-dimensional oxyselenide crystals
Source: Nat Commun. 2018 Aug 17;9:3311. doi: 10.1038/s41467-018-05874-2 (PMC6098096; doi:10.1038/s41467-018-05874-2)
Supplement: Supplementary file 1 — Supplementary Information [file 41467_2018_5874_MOESM1_ESM.pdf]

Supplementary Information

Ultrafast and highly-sensitive infrared photodetectors based on two-dimensional oxyselenide crystals

Authors: Jianbo Yin[1†], Zhenjun Tan[1,2†], Hao Hong[3†], Jinxiong Wu[1†], Hongtao Yuan[4], Yujing Liu[1], Cheng Chen[5], Congwei Tan[1], Fengrui Yao[3], Tianran Li[1], Yulin Chen[5], Zhongfan Liu[1,2], Kaihui Liu[2,3*], Hailin Peng[1,2*]

Affiliations:

[1] Center for Nanochemistry, Beijing Science and Engineering Centre for Nanocarbons, Beijing National Laboratory for Molecular Sciences, College of Chemistry and Molecular Engineering, Peking University, Beijing 100871, P. R. China

[2] Academy for Advanced Interdisciplinary Studies, Peking University, Beijing 100871, P. R. China

[3] State Key Laboratory for Mesoscopic Physics, School of Physics, Peking University, Beijing 100871, China

[4] National Laboratory of Solid-State Microstructures, College of Engineering and Applied Sciences, and Collaborative Innovation Center of Advanced Microstructures, Nanjing University, Nanjing 210093, P. R. China.

[5] Clarendon Laboratory, Department of Physics, University of Oxford, Parks Road, Oxford, OX1 3PU, UK

[†] These authors contributed equally to this work

*Correspondence: hlpeng@pku.edu.cn; khliu@pku.edu.cn

Supplementary Figures

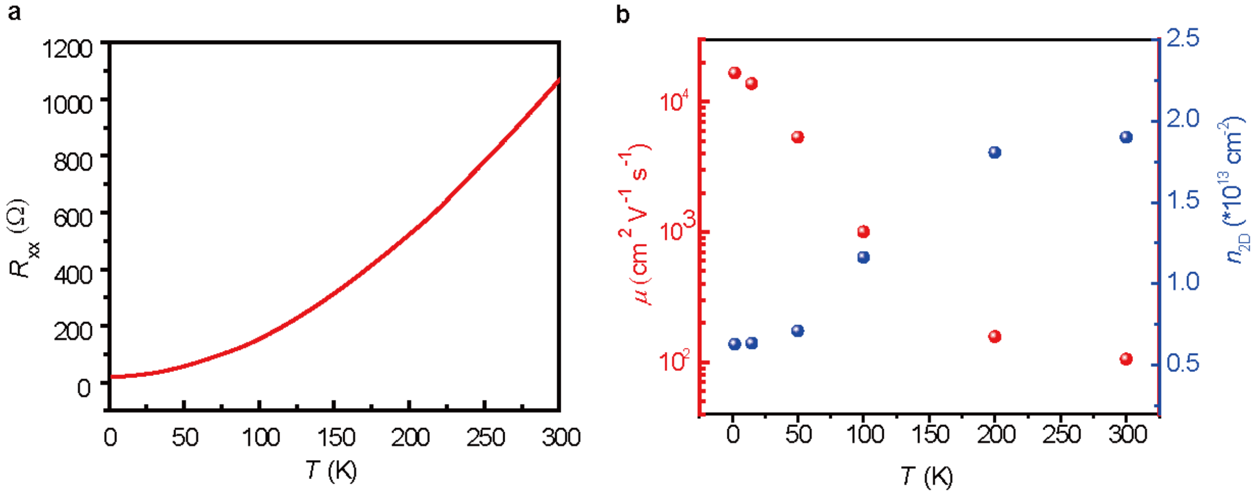

Supplementary Figure 1 | High mobility of 2D Bi_2O_2Se device. (a) Temperature dependence of longitudinal resistance in a typical Bi_2O_2Se device. (b) Temperature dependence of electron mobility. Left y axis shows mobility and right one shows carrier density with temperature.

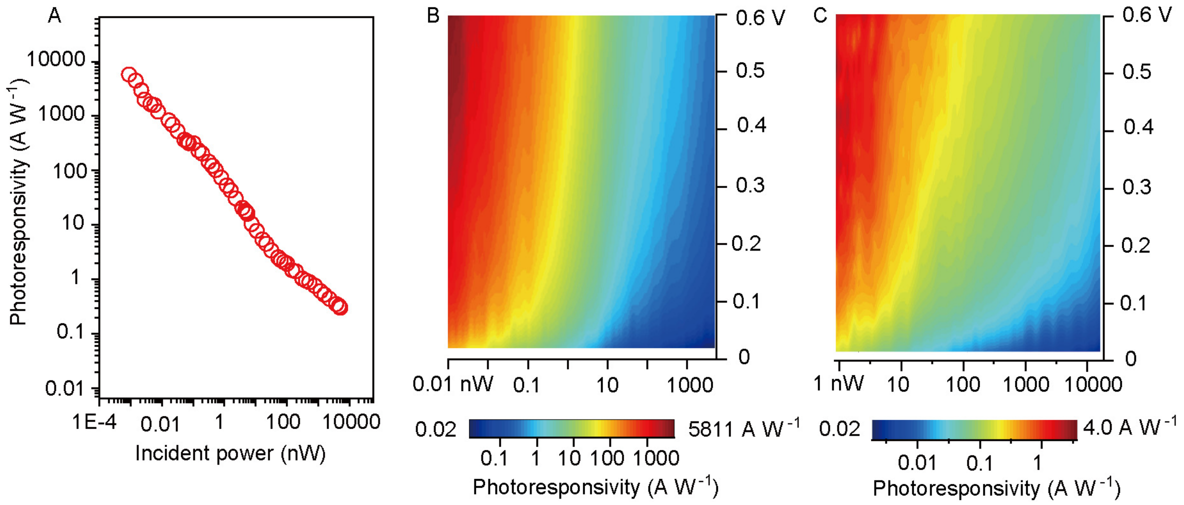

Supplementary Figure 2 | High photoresponsivity of 2D Bi_2O_2Se photodetector. (a) Photoresponsivity under 0.6 V bias at wavelength of 532 nm. (b) Dependence of photoresponsivity with incident power and voltage bias at 532 nm wavelength. (c) Dependence of photoresponsivity with incident power and voltage bias at 1310 nm wavelength.

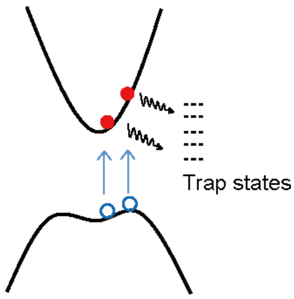

Supplementary Figure 3 | Illustration of trap states in photoconductive gain regime. Only electron trap states are shown here. Blue arrows show interband transitions, while black zigzag arrows show the trapping process.

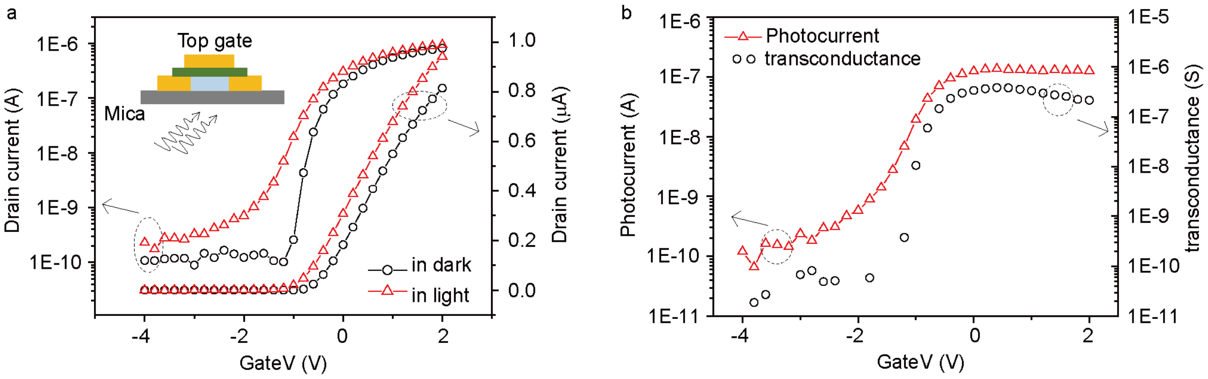

Supplementary Figure 4 | Similar trends between photocurrent and transconductance in photogating mechanism. (a) The transfer curves with and without light are shown by red and black circle, respectively. The left and right y-axis show linear and log scale. The device consists of approximately 10 nm thick Bi_2O_2Se on mica substrate with top gate structure, which has dielectric layer of 20 nm HfO_2 by ALD. (b) Gate-dependent photocurrent by triangle mark and transconductance by black circle.

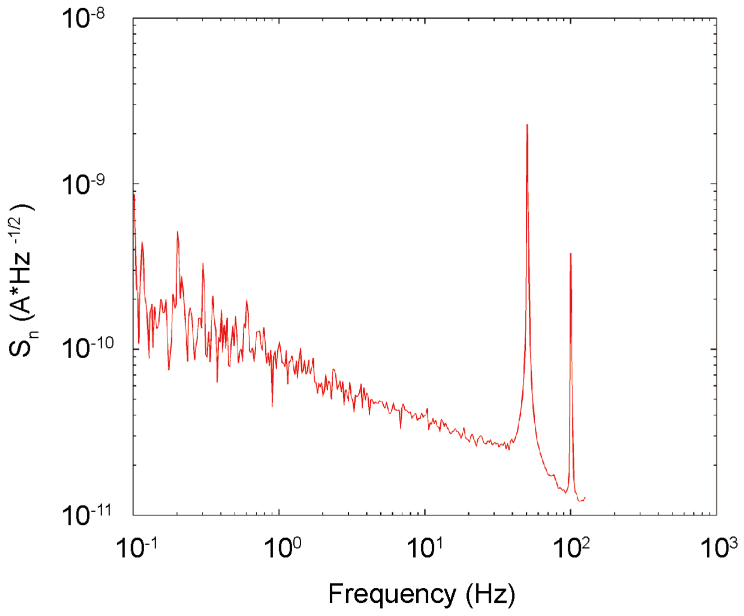

Supplementary Figure 5 | Noise spectrum of 2D Bi_2O_2Se photodetector. The noise shows a typical feature of 1/f noise with value of 8.5×10^{-11} A $Hz^{-1/2}$ at 1 Hz. The two peaks corresponds to 50 Hz and 100 Hz, which come from power line.

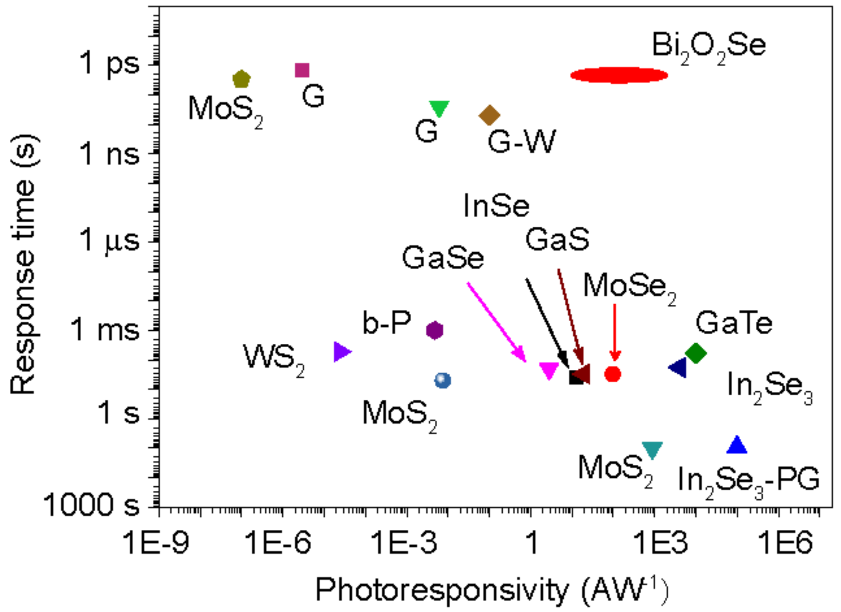

Supplementary Figure 6 | Photoresponse time of different 2D materials. From top to bottom: Intrinsic response time (photocarrier relaxing time) of graphene with approximately 1.5 ps[1,2] and Bi_2O_2Se with approximately 1 ps, graphene with 20 ps response time[3,4], graphene in waveguide (G-W)[5,6], Intrinsic response time (photocarrier relaxing time) of MoS_2[7], black phosphorus (bP)[8], WS_2[9], MoS_2[10], GaSe[11], InSe[12], GaS[13], $MoSe_2$[14], GaTe[15], In_2Se_3[16], MoS_2[17], In_2Se_3 in photogating mode[18.] We note that the response of bP, WS_2, GaSe, InSe, GaS, $MoSe_2$,

GaTe, In_2Se_3 are measured by turning on/off light, which are usually slower than that measured from pump-probe measurement with ultrafast laser.

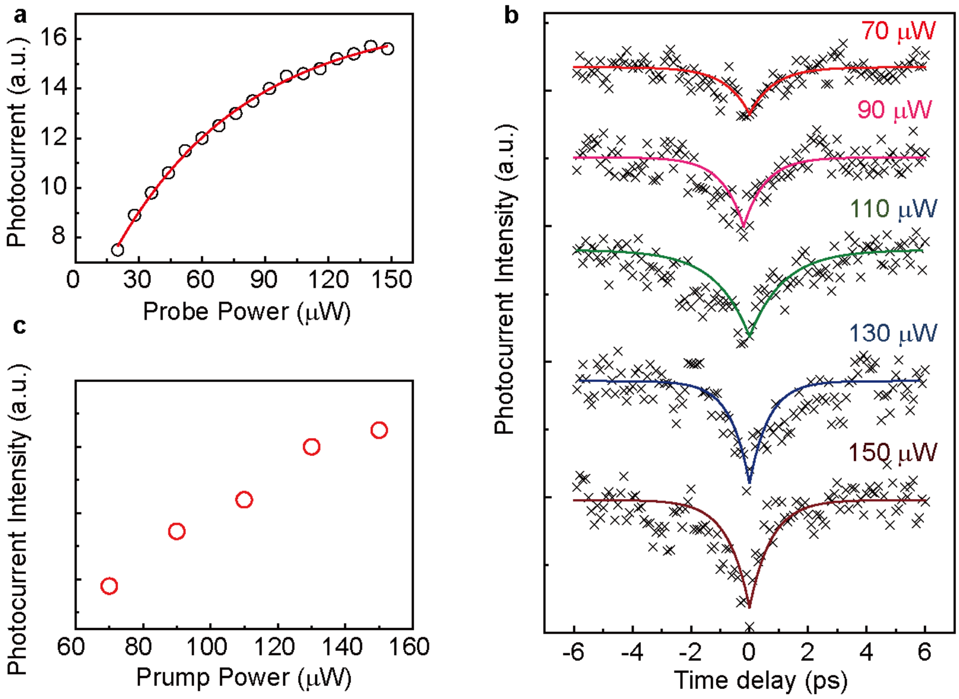

Supplementary Figure 7 | Time-resolved photocurrent spectroscopy of 2D Bi_2O_2Se photodetector. (a) Photocurrent as a function of laser power. Saturation absorption is observed when probe power reach 60 μW. (b) photocurrent intensity as a function of delay-time under different pump power. The probe power is fixed at 70 μW. The solid lines are fitted by exponential relation. The photocurrent decreased at zero delay. (c) The absolute values of the photocurrent obtained at time-zero with different pump power. The photocurrent at zero delay decreases as the pump power increases. These values show saturation as pump power increases, which is consistent with the saturation effect.

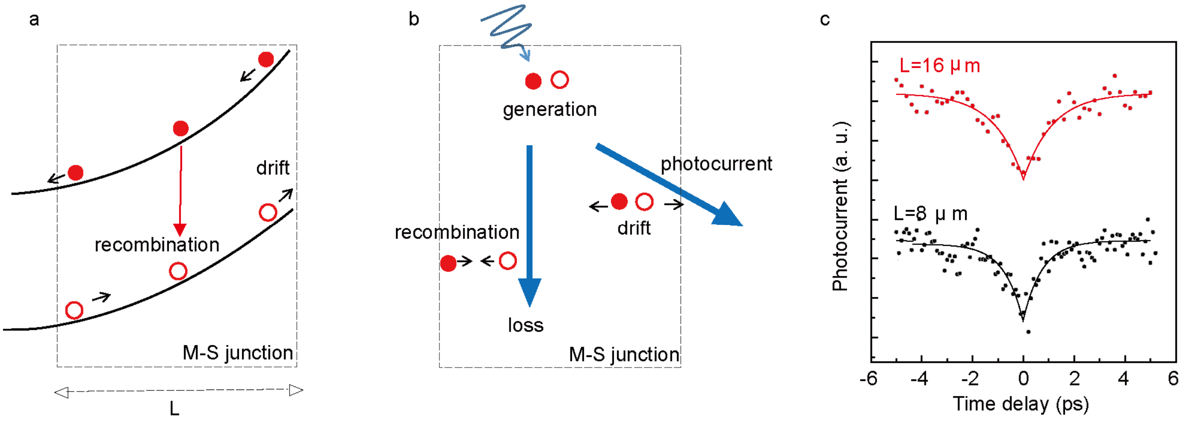

Supplementary Figure 8 | Photocarrier dynamic at metal-semiconductor junction. (a) Drift and recombination of photocarrier inside metal-semiconductor junction (M-S junction). Open and thick circles represent photoexited holes and photoexited electrons, respectively. Dashed rectangle indicates M-S junction with length of *L*. (b) Photocurrent generation and loss inside M-S junction. Photocarriers inside the M-S junction have two channels to decay. One is photocarrier drifting out of the junction which introduces photocurrent. The other one is electron-hole recombination which introduces loss. (c) Photoresponse times of Bi_2O_2Se device with channel lengths of 16 μm and 8 μm. The photoresponse times do not show channel-length dependence.

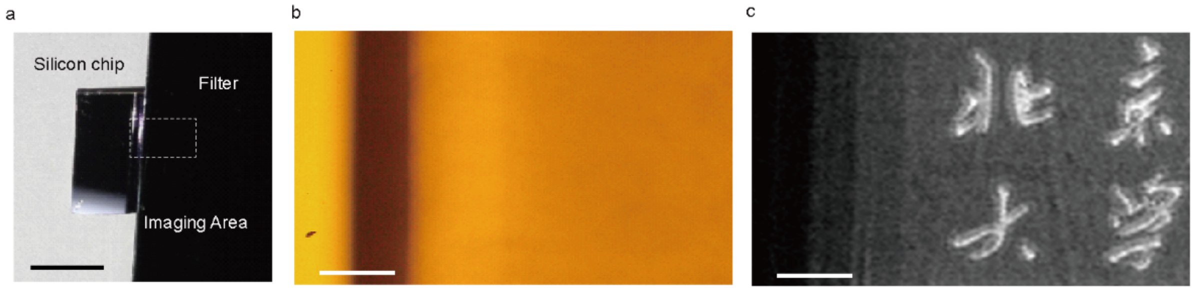

Supplementary Figure 9 | Infrared image taken by single pixel 2D Bi_2O_2Se. (a) Sample to be imaged. The sample consists of gold patterns (Chinese characters, not visible here) on silicon chip, which is covered by long-pass optical filter with cut-off wavelength of 780 nm. Scale bar is 5 mm. (b) Commercial camera picture of the imaging area in figure a. Since the imaging area is covered by the filter, the gold patterns are not observable here. Scale bar is 200 μm. (c) Infrared image taken by scanning focused 1150 nm beam over the same imaging area. The scheme of the imaging system is the same as shown in Fig. 4c. While scanning laser beam, a single pixel Bi_2O_2Se photodetector is used to detect the infrared reflecting signals at different locations. In contrast to commercial camera working in visible light range, the gold patterns could be easily observed by infrared Bi_2O_2Se photodetector. Scale bar is 200 μm.

Supplementary Note 1: Photogating effect in Bi_2O_2Se device

The high responsivity of Bi_2O_2Se device is attributed to the existence of photoconductive gain. Such gain mechanism could only prevail when there is an external source-drain bias. This photoconductive gain is usually introduced by localized states either inside 2D materials or at material-substrate interface. Under illumination, these localized states (namely trap states) could trap one type of photocarriers (electrons or holes), leaving the other type of photocarriers circulating as schematically shown in Supplementary Fig. 3. Therefore, this effect could prolong the lifetime (τ_{trap}) of the other type of carriers. If the lifetime τ_{trap} is larger than the transit time (τ_{transit}, the time of electron/hole travelling across the channel under source-drain bias), one photoexcitation event will introduce more than one electron transit event, which means the photoconductive gain ($^{\tau_{\mathrm{trap}}}/_{\tau_{\mathrm{transit}}}$) is larger than 1[19]. Such effect at some cases is also called photogating effect, as the localized charges at the trap states act like local gate, which effectively modulates a transistor behaviour, such as shifting the threshold voltage. This mechanism has been widely accepted in nanostructured materials—exacerbated by their high surface-to-volume ratio—such as colloid quantum dots[20], nanowires[21]. It has also been found and intensively studied in 2D materials, such as MoS_2[17,22] and black phosphorous[23].

To evidence the existence of photogating effect in Bi_2O_2Se device, we measured gate-dependent photocurrent by shedding light on the device. As shown in Supplementary Fig. 4a, the transfer curve under illumination shows a shift compared with the one in dark. This agrees well with the above-mentioned photogating effect, in which threshold voltage in transfer curve is shifted under illumination. To quantitatively study the effect, we calculated photocurrent and transconductance by equations:

$$I_{\mathrm{ph}} = I_{\mathrm{light}} - I_{\mathrm{dark}} \qquad (1)$$

$$g_{\mathrm{m}} = \frac{\Delta I_{\mathrm{dark}}}{\Delta V_{\mathrm{g}}} \qquad (2)$$

where I_{ph}, I_{ligh}, I_{dark}, g_{m} and V_{g} denote photocurrent, drain current under illumination, drain current in dark, the transconductance and gate voltage, respectively. As shown in Supplementary Fig. 4b, photocurrent and transconductance show similar trends, which agrees well with photogating theory[23], in which the photocurrent could be seen as drain current shift and follow the trend of transconductance through equation:

$$I_{\mathrm{ph}} = g_{\mathrm{m}} \times \Delta V_{\mathrm{th}} \qquad (3)$$

where ΔV_{th} is the threshold voltage shift. Based on the equation and data in Supplementary Fig. 4b, we extract ΔV_{th} =0.45 V. In this experiment, the similar trends between the photocurrent

and transconductance confirm the existence of photogating effect.

In addition, two very typical features of photogating effect were already shown in Fig. 2 in the main text. The first one is that responsivity decreases with incident power, which is shown in Fig 2a, 2b in main manuscript and Supplementary Fig. 2. The decrease is due to the limited density of state of trap states. As power increase, more photocarriers are excited, but similar amount of photocarrier can be trapped (thus relatively less), giving rise to lower responsivity. The second feature is the increasing sensitivity with increasing external bias as shown in Fig. 2b and the corresponding Supplementary Fig. 2b and 2c. As the gain ($^{\tau_{\mathrm{trap}}}/_{\tau_{\mathrm{transit}}}$) depends on transit time τ_{transit}, higher bias will shorten transit time and thus increase gain and responsivity.

In conclusion, the high responsivity in Bi_2O_2Se detector is due to photoconductive gain, which is introduced by trap states.

Supplementary Note 2: Ultrafast photoresponse measurement

In the measurement, two trains of ultrashort pulses (820 nm, 100 fs) with controllable time delay are focused on the same spot of metal-Bi_2O_2Se junction (M-S junction) in a device to excite photocarriers, which lead to photocurrent afterwards. This technique can probe photocarrier dynamic and photoresponse time by exploiting sublinear power dependence of photovoltage. Such sublinear behavior happens when shedding two trains of pulses with large power at the sample. During excitation, each pulse will excite photocarriers at the junction and then the two type of photocarriers drift separately due to build-in field at the Schottky barrier as shown in Supplementary Fig. 8a. If the two neighbour pulses from two beams are too temporally close, the photocarriers due to the first excitation does not have enough time to recombine, or escape (drift out of) the exciting area when the neighbour pulse arrives. These simultaneous excitations by the two neighbour pulses trigger photocurrent reduction and result in the lowest photocurrent when the two pulses fully overlap (as shown as dip at zero-time delay in Fig. 3a). In contrast, at long time delay, the photocarriers has enough time to either recombine, or escape (drift out of). The two pulses behave independently, resulting in a large photocurrent. Thus, by varying the delay time, we can extract the time duration from photocarrier generation to photocarrier disappearance at the junction (we neglect the photocarrier generation time as it is in general very fast[24]). This process is denoted as photocarrier decay, while the time duration is denoted as decay time in the following text.

Photocarriers inside the junction decay through two channels, which are recombination and drift as shown in Supplementary Fig. 8b (Note that we do not include exciton process here in order to simplify the explanation. If considering exciton processes such as exciton dissociation and recombination, we could treat exciton dissociation as part of the photocarrier drift process, while exciton recombination as a completely separate channel. The latter one usually has much longer time scale of approximately 1 ns[25-27]. This is much longer than free carrier recombination and thus does not affect our discussion here.). Total response time from the experiment is τ as revealed in Fig. 3, while photocarrier recombination time is τ_{recm}, and photocarrier drift time is τ_{drift}. The total decay rate is $1/\tau$, which denotes how fast the photocarriers decay. It is the adding up of the two decay rates $1/\tau_{\mathrm{recm}}$ and $1/\tau_{\mathrm{drift}}$ from the two channels. It follows equation as

$$\frac{1}{\tau} = \frac{1}{\tau_{\mathrm{recm}}} + \frac{1}{\tau_{\mathrm{drift}}} \quad (4)$$

The electron-hole recombination time τ_{recm} depends on the electron band structure, defect concentration, phonon band structure, surface states, etc. It is relatively long in semiconductors and was reported as more than 30 ps in transition metal dichalcogenide (TMD)[25,28]. Therefore, we believe it is the faster decay channel—drift channel—dominate the whole decay process, although the domination of recombination channel cannot be completely ruled out here. The drift process does strongly depend on the mobility. For this channel the drifting time can be calculated by equation[29]

$$\tau_{\mathrm{drift}} = \frac{L^2}{2\mu V} \qquad (5)$$

where L, μ, V are length of M-S junction, carrier mobility, and Schottky barrier height, respectively. Based on Supplementary equation 5, τ_{drift} depends on length of M-S junction, instead of the whole channel length. This is because as soon as one type of photocarriers drifts out of the junction, there will be charge accumulation and then photocurrent in the circuit. To confirm this theory, we conducted photoresponse measurement for devices with different channel-lengths. As expected, they do not lead to different photoresponse time. Instead, both devices with channel lengths of 16 μm and 8 μm show similar photoresponse time of approximately 1 ps (Supplementary Fig. 8c), which agrees well with that the drift time and photoresponse time relates to length of the junction instead of the channel length.

Supplementary Note 3: Chemical etching process with facile region-selectivity

CVD-grown 2D Bi_2O_2Se crystals on mica substrates can be patterned into ordered arrays by a facile wet-chemical etching method using dilute H_2O_2 and protonic acid as mixed etchants. Assistant by microfabrication technique and pre-patterned lithography mask, the pre-designed patterns can be realized by standard electron beam lithography (EBL) process. Then the optimized component ratio of H_2O_2: H_3O^+: H_2O at 2:4:8 can facilitate the facile chemical patterning of 2D Bi_2O_2Se crystals. A typical region-selective chemical etching process can be completed at approximately 20 s and then the samples should be quickly transferred to large quantities of water and rinsed for several times to terminate the etching reaction and avoid end-cutting effect. Finally, ordered 2D Bi_2O_2Se arrays can be obtained after the removal of patterned mask.

Supplementary References

1. Tielrooij, K. J., Song, J. C. W., Jensen, S. A., Centeno, A., Pesquera, A., Elorza, A. Z., Bonn, M., Levitov, L. S., Koppens, F. H. L. Photoexcitation cascade and multiple hot-carrier generation in graphene. *Nat. Phys.* 9, 248-252 (2013).
2. Sun, D., Aivazian, G., Jones, A. M., Ross, J. S., Yao, W., Cobden, D., Xu, X. D. Ultrafast hot-carrier-dominated photocurrent in graphene. *Nat. Nanotechnol.* 7, 114-118 (2012).
3. Xia, F. N., Mueller, T., Lin, Y. M., Valdes-Garcia, A., Avouris, P. Ultrafast graphene photodetector. *Nat. Nanotechnol.* 4, 839-843 (2009).
4. Mueller, T., Xia, F., Avouris, P. Graphene photodetectors for high-speed optical communications. *Nat. Photon.* 4, 297-301 (2010).
5. Gan, X., Shiue, R. J., Gao, Y., Meric, I., Heinz, T. F., Shepard, K., Hone, J., Assefa, S., Englund, D. Chip-integrated ultrafast graphene photodetector with high responsivity. *Nat. Photon.* 7. 883-887 (2013).
6. Pospischil, A., Humer, M., Furchi, M. M., Bachmann, D., Guider, R., Fromherz, T., Mueller, T. CMOS-compatible graphene photodetector covering all optical communication bands. *Nat. Photon.* 7, 892-896 (2013).
7. Wang, H., Zhang, C., Chan, W., Tiwari, S., Rana, F. Ultrafast response of monolayer molybdenum disulfide photodetectors. *Nat. Commun.* 6, 8831 (2015).
8. Buscema, M., Groenendijk, D. J., Blanter, S. I., Steele, G. A., Van Der Zant, H. S., Castellanos-Gomez, A. Fast and Broadband Photoresponse of Few-Layer Black Phosphorus Field-Effect Transistors. *Nano Lett.* 14, 3347-3352 (2014).

9. Perea-Lopez, N., Elías, A. L., Berkdemir, A., Castro-Beltran, A., Gutierrez, H. R., Feng, S., Lv, R., Hayashi, T., Lopez-Urias, F., Ghosh, S., Muchharla, B., Talapatra, S., Terrones, H., Terrones, M. Photosensor Device Based on Few-Layered WS_2 Films. *Adv. Funct. Mater.* 23, 5511-5517 (2013).
10. Yin, Z., Li, H., Li, H., Jiang, L., Shi, Y., Sun, Y., Lu, G., Zhang, Q., Chen, X., Zhang, H. Single-Layer MoS_2 Phototransistors. *ACS Nano* 6, 74-81 (2012).
11. Hu, P., Wen, Z., Wang, L., Tan, P., Xiao, K. Synthesis of Few-Layer GaSe Nanosheets for High Performance Photodetectors. *ACS Nano* 6, 5988-5994 (2012).
12. Tamalampudi, S. R., Lu, Y. Y., Kumar, U, R., Sankar, R., Liao, C. D., Moorthy, B, K., Cheng, C. H., Chou, F. C., Chen, Y. T. High Performance and Bendable Few-Layered InSe Photodetectors with Broad Spectral Response. *Nano Lett.* 14, 2800-2806 (2014).
13. Hu, P., Wang, L., Yoon, M., Zhang, J., Feng, W., Wang, X., Wen, Z. Z., Idrobo, J. C., Miyamoto, Y., Geohegan, D. B., Xiao, K. Highly Responsive Ultrathin GaS Nanosheet Photodetectors on Rigid and Flexible Substrates. *Nano Lett.* 13, 1649-1654 (2013).
14. Abderrahmane, A., Ko, P. J., Thu, T. V., Ishizawa, S., Takamura, T., Sandhu, A. High photosensitivity few-layered $MoSe_2$ back-gated field-effect phototransistors. *Nanotechnology*, 25, 365202 (2014).
15. Liu, F., Shimotani, H., Shang, H., Kanagasekaran, T., Zolyomi, V., Drummond, N., Fal'ko, V. I., Tanigaki, K. High-Sensitivity Photodetectors Based on Multilayer GaTe Flakes. *ACS Nano* 8, 752-760 (2014).
16. Jacobs-Gedrim, R. B., Shanmugam, M., Jain, N., Durcan, C. A., Murphy, M. T., Murray, T. M., Matyi, R. J., Moore, R. L., Yu, B. Extraordinary Photoresponse in Two-Dimensional In_2Se_3 Nanosheets. *ACS Nano* 8, 514-521 (2014).
17. Lopez-Sanchez, O., Lembke, D., Kayci, M., Radenovic, A., Kis, A. Ultrasensitive photodetectors based on monolayer MoS_2. *Nat. Nanotechnol.* 8, 497-501 (2013).
18. Island, J. O., Blanter, S. I., Buscema, M., van der Zant, H. S., Castellanos-Gomez, A. Gate Controlled Photocurrent Generation Mechanisms in High-Gain In_2Se_3 Phototransistors. *Nano Lett.* 15, 7853-7858 (2015).
19. Yariv, A. Optical electronics in modern communications; Oxford University Press, 5th edition, 425-427, 1997.
20. Konstantatos, G., Badioli, M., Gaudreau, L., Osmond, J., Bernechea, M., De Arquer, F. P. G., Gatti, F., Koppens, F. H. L. Hybrid graphene-quantum dot phototransistors with ultrahigh gain. *Nat. Nanotechnol.* 7, 363-368 (2012).

21. Soci, C., Zhang, A., Bao, X. Y., Kim, H., Lo, Y., Wang, D. Nanowire photodetectors. *J. Nanosci Nanotechno*. 10, 1430-1449 (2010).
22. Furchi, M. M., Polyushkin, D. K., Pospischil, A., Mueller, T. Mechanisms of photoconductivity in atomically thin MoS_2. *Nano Lett.* 14, 6165-6170 (2014).
23. Guo, Q., Pospischil, A., Bhuiyan, M., Jiang, H., Tian, H., Farmer, D., Deng, B., Li, C., Han, S., Wang, H., Xia, Q., Ma, T., Mueller, T., Xia, F. Black phosphorus mid-infrared photodetectors with high gain. *Nano Lett.* 16, 4648-4655 (2016).
24. Tielrooij, K. J., Piatkowski, L., Massicotte, M., Woessner, A., Ma, Q., Lee, Y., Myhro, K. S., Lau, C. N., Jarillo-Herrero, P., van Hulst, N. F., Koppens, F. H. L. Generation of photovoltage in graphene on a femtosecond timescale through efficient carrier heating. *Nat. Nanotechnol.* 10, 437 (2015).
25. Massicotte, M., Vialla, F., Schmidt, P., Lundeberg, M. B., Latini, S., Haastrup, S., Danovich, M., Davydovskaya, D., Watanabe, K., Taniguchi, T., Fal'ko, V. I., Thygesen, K. S., Pedersen, T. G., Koppens, F. H. L. Dissociation of two-dimensional excitons in monolayer WSe_2. *Nat. Commun.* 9, 1633 (2018).
26. Mouri, S., Miyauchi, Y., Toh, M., Zhao, W., Eda, G., Matsuda, K. Nonlinear photoluminescence in atomically thin layered WSe_2 arising from diffusion-assisted exciton-exciton annihilation. *Phys. Rev. B.* 90, 155449 (2014).
27. Yuan, L., Huang, L. Exciton dynamics and annihilation in WS_2 2D semiconductors. *Nanoscale* 7, 7402-7408 (2015).
28. Aivazian, G., Yu, H., Wu, S., Yan, J., Mandrus, D. G., Cobden, D., Yao, W., Xu, X. Many-body effects in nonlinear optical responses of 2D layered semiconductors. *2D Materials* 4, 025024 (2017).
29. Gabor, N. M., Zhong, Z., Bosnick, K., McEuen, P. L. Ultrafast photocurrent measurement of the escape time of electrons and holes from carbon nanotube p−i−n photodiodes. *Phys. Rev. Lett.* 108, 087404 (2012).
