# Supplementary material for: Ultrafast and highly sensitive infrared photodetectors based on two-dimensional oxyselenide crystals
Source: Nat Commun. 2018 Aug 17;9:3311. doi: 10.1038/s41467-018-05874-2 (PMC6098096; doi:10.1038/s41467-018-05874-2)
Supplement: Supplementary file 2 — Description of Additional Supplementary Files [file 41467_2018_5874_MOESM2_ESM.pdf]

Description of Additional Supplementary Files

File Name: Supplementary Movie 1
Description: Scanning an infrared image by a single Bi_2O_2Se photodetector. The sample consists of gold pattern on silicon chip, which is under 1150 nm laser illumination. Sample size is in scale of 200 µm.
